# Advanced strategies for the removal of venlafaxine from aqueous environments: a critical review of adsorption and advanced oxidation pathways

**DOI:** 10.1039/d5ra06996c

**Published:** 2025-10-16

**Authors:** Harez Rashid Ahmed, Dlzar D. Ghafoor, Nian N. M. Agha, Gasha Abdullah Muhamad, Pavel Husamadin, Tariq Muhammad Ali

**Affiliations:** a Department of Chemistry, College of Science, University of Sulaimani, Kurdistan Regional Government Qlyasan Street Sulaymaniyah 46001 Iraq harez.ahmed@univsul.edu.iq; b College of Science, Department of Biomedical, Komar University of Science and Technology Sulaimani 46001 Iraq; c Department of Pharmacy, Kurdistan Technical Institute Sulaimani 401 KGR/Iraq; d College of Science, Department of Medical Laboratory Science, Komar University of Science and Technology Sulaimani 46001 Iraq; e College of Pharmacy, Department of Pharmacy, Al-Esraa University Baghdad Baghdad Governorate Iraq

## Abstract

The persistence of venlafaxine (VEN), a widely prescribed antidepressant [1-[2-(dimethylamino)-1-(4-methoxyphenyl)ethyl]cyclohexan-1-ol], in aquatic environments raises serious concerns due to its chemical stability, bioaccumulation potential, and ecotoxicological impacts. Conventional wastewater treatment technologies are largely ineffective in eliminating such micropollutants, achieving removal efficiencies typically below 30%. This review critically evaluates and compares adsorption-based and advanced oxidation process (AOP)-based strategies for VEN removal from aqueous systems. Particular attention is given to adsorption mechanisms hydrophobic, π–π, electrostatic, and hydrogen-bonding interactions, using biochar, activated carbon, and emerging nanomaterials. Concurrently, state-of-the-art AOPs, including Fenton-like, photocatalytic, and persulfate-based oxidation, are analyzed with emphasis on radical generation, catalyst design, and operational optimization. Integrating heterogeneous catalysts such as metal oxide nanocomposites and graphene-based frameworks significantly enhances degradation kinetics, selectivity, and reusability under mild reaction conditions. Comparative analysis reveals that adsorption provides an eco-friendly, cost-effective pre-treatment, while AOPs enable complete mineralization and are ideal as polishing steps in hybrid treatment systems. This review offers the first comprehensive comparison of adsorption and advanced oxidation pathways for VEN removal, incorporating recent advances (2022–2024) and providing mechanistic insights, efficiency trends, and sustainability perspectives to guide future research on green wastewater remediation.

## Abstract 2

This review compares adsorption and advanced oxidation pathways for removing venlafaxine from water, highlighting mechanisms, catalyst design, and sustainability in modern wastewater treatment.

## Graphical abstract

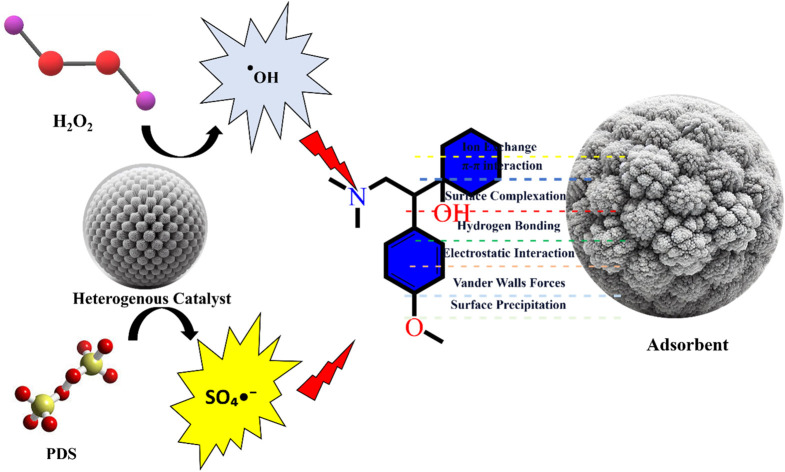

## 1 Introduction

While essential for human and animal health, pharmaceutical compounds are increasingly recognized as emerging environmental pollutants due to their persistence and bioactivity in natural ecosystems.^1,2^ These substances enter aquatic environments primarily through wastewater effluents from domestic sewage, pharmaceutical manufacturing, hospital discharge, veterinary applications, and, in some cases, improper disposal such as flushing unused medications down toilets. Conventional municipal wastewater treatment plants (WWTPs), which are designed to remove macro pollutants and pathogens, are typically ineffective in eliminating these trace-level contaminants. As a result, pharmaceuticals and their metabolites, often excreted unmetabolized or as biologically active compounds, remain in treated effluents and are continuously introduced into surface and groundwater systems.

Among the various pharmaceutical contaminants, antidepressants, particularly selective serotonin-norepinephrine reuptake inhibitors (SNRIs), are gaining significant attention. These compounds are extensively prescribed and have demonstrated biological activity even at nanogram per liter concentrations, posing ecotoxicological risks to aquatic organisms.^3–7^ Venlafaxine (VEN), a prominent SNRI, is widely used to treat major depressive disorder, generalized anxiety, social phobia, and panic disorders. Additionally, it is often prescribed off-label for conditions such as fibromyalgia, diabetic neuropathy, post-traumatic stress disorder, and migraine prevention. Mechanistically, venlafaxine acts by inhibiting serotonin and norepinephrine reuptake at presynaptic terminals, thus enhancing neurotransmission in the central nervous system.^8–10^ The pharmacological action is highly selective and does not significantly affect other neurotransmitter pathways, contributing to its clinical efficacy and widespread usage.

However, the widespread consumption of venlafaxine has led to its frequent detection in wastewater effluents and natural water bodies. Its bicyclic phenylethylamine derivative's chemical structure confers high stability, low biodegradability, and resistance to conventional biological treatment processes. Consequently, venlafaxine has been classified as a high-priority emerging contaminant due to its environmental persistence and potential for bioaccumulation. Even at low ecological concentrations, chronic exposure has been associated with behavioral and physiological disturbances in aquatic species, particularly fish and invertebrates, through interference with neurotransmitter systems.^11–13^

To address these environmental concerns, advanced physicochemical treatment technologies have been investigated to remove venlafaxine from aqueous media. Among these, adsorption and degradation-based approaches have emerged as promising solutions. Adsorption technologies, utilizing materials such as activated carbon, biochar, graphene oxide, and metal–organic frameworks, leverage their high surface area and tunable chemical functionalities to capture venlafaxine molecules efficiently. Meanwhile, advanced oxidation processes (AOPs), including photocatalysis, ozonation, Fenton and Fenton-like systems, and sulfate radical-based oxidation, rely on the generation of highly reactive species (*e.g.*, hydroxyl and sulfate radicals) to degrade venlafaxine into less toxic or mineralized forms.^4,14–18^ Several techniques have been explored for removing venlafaxine (VEN) from aqueous environments, including membrane filtration, biological treatment, photolysis, adsorption, and advanced oxidation processes (AOPs). Among these, adsorption and degradation-based methods, particularly AOPs, have emerged as the most promising due to their high removal efficiency, environmental compatibility, and adaptability to various water matrices. Adsorption is favored for its simplicity, cost-effectiveness, and lack of harmful byproducts, while AOPs are recognized for their ability to mineralize VEN into harmless end-products such as CO_2_ and H_2_O.

Despite encouraging results, existing literature on these removal methods remains fragmented, and a holistic understanding of their comparative performance, removal mechanisms, limitations, and long-term sustainability is still lacking. Therefore, this review aims to provide a comprehensive and critical analysis of current research on venlafaxine remediation strategies. The article specifically focuses on (i) adsorption mechanisms and material interactions, (ii) radical-driven degradation pathways, and (iii) comparative evaluation of these two classes of treatment technologies. In addition, the review discusses the formation and toxicity of transformation products (TPs), operational challenges, and the future direction of integrated or hybrid systems for more effective and environmentally sound removal of venlafaxine from water systems.

In contrast to earlier reviews that broadly examined antidepressant removal or general pharmaceutical wastewater treatment, this review offers a distinctive and critical synthesis focused exclusively on venlafaxine (VEN), a persistent and frequently detected antidepressant in aquatic environments. It systematically compares adsorption-based and advanced oxidation process (AOP) based techniques, elucidating both mechanistic and performance-oriented perspectives. The novelty of this work lies in integrating recent research advances (2022–2024) to analyze removal efficiencies, degradation intermediates, and regeneration behaviors of adsorbents and catalysts. Furthermore, this review identifies current limitations, highlights sustainability challenges, and proposes hybrid treatment strategies combining adsorption and oxidation to achieve complete mineralization of VEN with minimal environmental impact. Such a comprehensive and up-to-date evaluation has not yet been reported in the existing literature and aims to inform the design of efficient, eco-friendly wastewater treatment technologies for the future.^19,20^

## 2 Physicochemical properties and environmental behavior of venlafaxine

Venlafaxine is a synthetic tertiary amino compound structurally characterized by the presence of an *N*,*N*-dimethyl-ethanamine backbone substituted at the first position with a 1-hydroxycyclohexyl group and a 4-methoxyphenyl moiety. As a member of the selective serotonin-norepinephrine reuptake inhibitor (SNRI) class, venlafaxine functions pharmacologically as an antidepressant, serotonin uptake inhibitor, adrenergic and dopaminergic uptake inhibitor, and analgesic. It is also categorized as an emerging environmental contaminant and xenobiotic.^21^ Clinically, venlafaxine is widely prescribed for managing major depressive disorder, generalized anxiety disorder, panic disorder, and other off-label uses. However, its increasing global consumption, poor metabolism, and low environmental biodegradability have led to its frequent detection in aquatic systems. These properties raise significant ecotoxicological concerns due to their persistence and biological activity at trace levels.^22^

### 2.1 Physicochemical properties of venlafaxine

Venlafaxine exhibits high polarity and substantial water solubility (566 mg L^−1^ at 25 °C), facilitating its transport in aquatic environments. With a molecular weight of 277.4 g mol^−1^ and a p*K*_a_ of 9.4, venlafaxine predominantly exists in a protonated form under environmental pH conditions, which affects its environmental partitioning and chemical behavior. Its log *K*_ow_ value of 3.2 indicates moderate hydrophobicity and potential for bioaccumulation. These properties are summarized in Table 1.^23,24^

**Table 1:** Physicochemical properties of venlafaxine relevant to its environmental fate

Properties	Value
Molecular formula	C_17_H_27_NO_2_
Molecular weight	277.4 g mol^−1^
Water solubility	566 mg L^−1^ at 25 °C
p*K*_a_	9.4
Vapor pressure	3.64 × 10^−6^ Pa
Biodegradability	Poor

The methoxy and cyclohexanol functional groups within the venlafaxine molecule contribute to its environmental persistence by resisting microbial degradation. At the same time, the amine moiety enhances interactions with adsorbent surfaces and oxidative agents.^25,26^

### 2.2 Environmental occurrence and fate

Venlafaxine has been detected in various environmental matrices across North America, Europe, and Asia, including municipal wastewater, surface waters, groundwater, and even treated drinking water. Venlafaxine (VEN) has been detected in multiple environmental matrices across North America, Europe, and Asia, including municipal wastewater, surface waters, groundwater, and even treated drinking water.^27^ Its primary environmental release pathways can be categorized as follows: (1) incomplete removal during wastewater treatment processes, where conventional biological treatments fail to fully degrade VEN and its metabolites, leading to their continuous discharge into effluents. (2) Human excretion of unmetabolized venlafaxine and its primary active metabolite, *O*-desmethylvenlafaxine (ODV), which enter domestic sewage systems through urine and feces after therapeutic use. This pathway represents a direct source of pharmaceutical residues to wastewater treatment plants. (3) Improper disposal of unused or expired medications, which may be flushed into sewage or disposed of with household waste, leading to leaching into groundwater or surface water.^28–31^

Following administration, a considerable portion of venlafaxine and ODV is excreted through urine and feces, subsequently entering wastewater streams. Following administration, a significant portion of venlafaxine and ODV is excreted through urine and feces, subsequently entering wastewater streams.^32^ In addition, improper disposal, such as flushing unused drugs down toilets or discarding them with household waste, contributes to environmental contamination WHO, 2012. Due to its weak sorption to sludge and sediment particles, venlafaxine remains predominantly in the dissolved phase, increasing its mobility and persistence in aquatic ecosystems.^33^

Concentrations of venlafaxine reported in WWTP effluents range from 10 to 8000 ng L^−1^, with surface water levels reaching up to 1000 ng L^−1^.^34,35^ In some cases, exceptionally high concentrations have been reported up to 2.19 g L^−1^ in municipal wastewater and 1.31 g L^−1^ in effluent-dominated streams. Locally, in the Speed River, venlafaxine and *O*-desmethylvenlafaxine were detected downstream from a tertiary sewage treatment facility at 0.253 g L^−1^ and 0.486 g L^−1^, respectively.^36^

### 2.3 Ecotoxicological impact

Even at sub-ng L^−1^ concentrations, venlafaxine poses chronic risks to aquatic life, particularly fish and invertebrates. Its pharmacological activity interferes with neurotransmitter function, altering behaviors such as reproduction, predator avoidance, and physiological stress responses.^37–39^ Conventional wastewater treatment plants are not designed to remove micropollutants, allowing venlafaxine and its metabolites to persist in effluents and receiving water bodies.

**Fig. 1:**
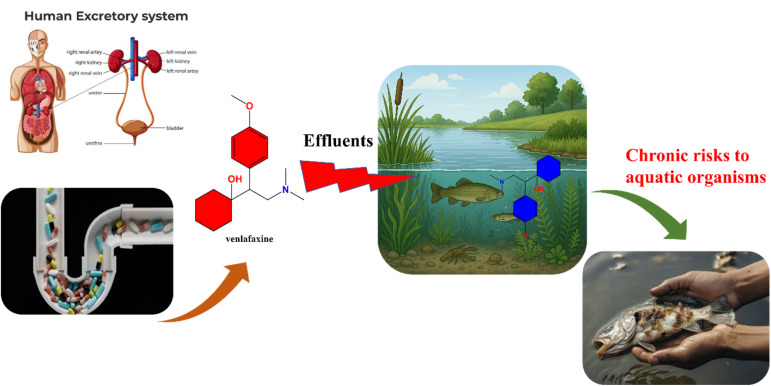
Environmental pathways and ecotoxicological risks of venlafaxine and its transformation products.

Furthermore, transformation products, including *O*-desmethylvenlafaxine and various hydroxylated or oxidized intermediates, may possess toxicity levels comparable to or greater than the parent compound. However, these transformation products' environmental behavior, fate, and ecotoxicity are still insufficiently characterized, necessitating further investigation.^40,41^ Venlafaxine (VEN) and its active metabolite *O*-desmethylvenlafaxine (ODV) have been reported to induce chronic toxic effects in aquatic organisms, even at environmentally relevant concentrations. Prolonged exposure has been linked to neurobehavioral disorders, endocrine disruption, and oxidative stress in fish and invertebrates. For example, chronic exposure (≥10 μg L^−1^) caused altered swimming activity, feeding inhibition, and anxiety-like behavior in *Danio rerio* due to serotonin reuptake interference.^42–44^ Moreover, reproductive impairments, including reduced fecundity and delayed embryonic development, have been observed in *Oryzias latipes* and *Pimephales promelas* exposed to VEN-contaminated waters.^45^ VEN also induces oxidative stress and enzyme inhibition in *Daphnia magna* and *Chironomus riparius*, indicating potential risks to aquatic food webs.^46^ These chronic effects underline the ecotoxicological significance of VEN contamination, supporting the urgent need for efficient removal strategies through adsorption and advanced oxidation processes. Fig. 1 illustrates the environmental exposure routes and associated ecotoxicological risks of venlafaxine in aquatic systems.

## 3 Adsorption-based removal techniques

Adsorption has emerged as a highly efficient and environmentally friendly technique for removing pharmaceutical contaminants such as venlafaxine (VEN) from aquatic systems. Due to its simplicity, low operational cost, and adaptability across various treatment configurations, adsorption is a promising solution for tackling persistent micro-pollutants. The accumulation of solutes governs this physicochemical process, VEN, at the interface between a liquid phase (wastewater) and a solid phase (adsorbent), making it particularly suitable for water purification applications.

The effectiveness of VEN adsorption depends mainly on its structural and chemical attributes, including its amphipathic nature, moderate hydrophobicity (log *K*_ow_ ≈ 3.2), tertiary amine functionality, and aromaticity. These properties allow VEN to interact with various adsorbent materials through multiple mechanisms. Traditionally, activated carbon has been the most commonly used adsorbent due to its high surface area and tunable surface chemistry. However, the development of alternative adsorbents has garnered increasing interest in recent years. These include natural and engineered materials such as clay minerals, agricultural and industrial biomass residues, zeolites, and various carbonaceous materials.^47^ In particular, biochar, produced through the pyrolysis of organic waste, has received significant attention as a sustainable and low-cost adsorbent. Its utilization aligns with circular economy principles, converting organic waste into value-added products while contributing to environmental remediation. Numerous studies have demonstrated the high adsorption capacity of biochar toward pharmaceutical residues, including VEN.^48–50^ Despite these advantages, most current research focuses on batch systems targeting single-contaminant solutions. A significant gap remains in the literature regarding adsorption under realistic conditions, such as multi-contaminant systems and continuous flow operations.^48^

### 3.1 Overview of adsorption mechanism

The adsorption of VEN onto various materials involves a complex interplay of physicochemical interactions strongly influenced by environmental conditions and adsorbent surface characteristics. The primary mechanisms include.

#### 3.1.1 Hydrophobic interactions

VEN exhibits moderate hydrophobicity, enabling it to associate with hydrophobic regions of adsorbents. This is particularly relevant for nonpolar or carbon-based surfaces. Hydrogen bonding between hydroxyl groups on the adsorbent and the polar functional groups on VEN can also facilitate these interactions.^51^

#### 3.1.2 π–π interactions

The aromatic moiety in VEN enables π–π stacking interactions with the delocalized π-electron systems of carbonaceous adsorbents such as graphene oxide, carbon nanotubes, and activated carbon.

#### 3.1.3 Electrostatic interactions and ion exchange

Depending on the adsorbent's solution pH and the isoelectric point (pH_ZPC), VEN may interact electrostatically in its protonated or neutral form. In acidic conditions, VEN predominantly exists in a cationic state (due to its p*K*_a_ ≈ of approximately 9.4), which promotes electrostatic attraction with negatively charged adsorbent surfaces. Additionally, ion exchange processes may occur between the functional groups on VEN (*e.g.*, amine) and the ionizable groups (*e.g.*, –OH, –COOH) on the adsorbent.^51^

#### 3.1.4 Hydrogen bonding

VEN's hydroxyl and amino functional groups can form strong hydrogen bonds with polar groups on the adsorbent surface. This mechanism is crucial for oxygen-rich adsorbents, such as biochar, cellulose, or silica-based materials.

#### 3.1.5 Pore filling

Adsorption can also occur through the physical entrapment of VEN molecules within the adsorbent's mesoporous or microporous structure. The high surface area and well-developed porosity enhance the capacity for such pore-filling mechanisms.

#### 3.1.6 Surface complexation

Surface complexation refers to the chemisorption of VEN through interactions with active surface sites, particularly those involving metal oxides or functional groups that bear transition metals. These interactions often involve multidentate binding and can significantly influence removal efficiency.^52^

Environmental variables such as pH, temperature, ionic strength, and the presence of competing organic or inorganic species play critical roles in modulating these adsorption pathways. For example, increasing temperature may enhance diffusion rates and overall adsorption kinetics, while competing ions can inhibit electrostatic binding or ion exchange.

**Fig. 2:**
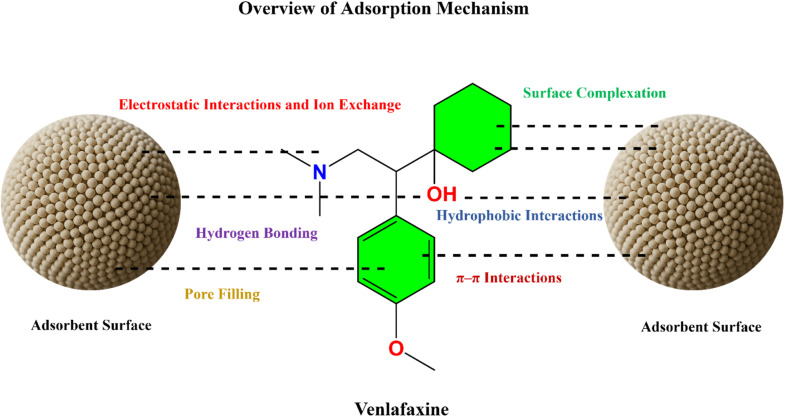
Environmental pathways and ecotoxicological risks of venlafaxine and its transformation products.

Fig. 2 comprehensively illustrates these interaction mechanisms, offering visual insight into how VEN engages with different surface functionalities and adsorbent morphologies under varied environmental conditions.

### 3.2 Adsorbents used for venlafaxine removal

The adsorption process using solid-phase materials has emerged as one of the most promising and widely researched techniques for remediating water and wastewater contaminated with pharmaceutical compounds.^53^ This approach is particularly valued for its cost-effectiveness, operational simplicity, environmental sustainability, and ability to achieve high removal efficiencies, even at trace concentrations of contaminants. Among pharmaceutical pollutants, venlafaxine (VEN), a serotonin-norepinephrine reuptake inhibitor frequently detected in effluents from municipal wastewater treatment plants, has garnered significant attention due to its persistence and potential ecotoxicological effects.

Adsorbents used for treating pharmaceutical-laden waters are generally classified into natural and synthetic materials, each possessing distinctive physicochemical characteristics such as porosity, specific surface area, surface functional groups, and structural stability. Natural adsorbents encompass activated charcoal, clays, zeolites, and mineral ores. These materials are widely available, low-cost, and offer promising adsorption capabilities, especially when subjected to surface modifications to enhance their affinity for target pollutants. On the other hand, synthetic adsorbents are often derived from a broad range of precursor materials, including agricultural residues, industrial byproducts, polymeric compounds, and municipal solid wastes. A wide variety of raw materials, such as coconut shells, fruit peels, date seeds, rice husks, sawdust, chitosan, sugarcane bagasse, fly ash, red mud, tire-derived carbon, seafood shell waste, and industrial slags have been successfully converted into functional adsorbents through pyrolysis, chemical activation, or surface functionalization.^54,55^

The valorization of such waste materials aligns with circular economy principles, transforming them into practical tools for environmental remediation.

Several specific adsorbents have been evaluated for their effectiveness in removing VEN from aqueous solutions, each offering unique mechanisms and affinities.

#### 3.2.1 Activated carbon (AC)

Activated carbon remains the benchmark adsorbent in water treatment due to its exceptional surface area, high microporosity, and versatile surface chemistry. It has demonstrated high affinity toward various pharmaceuticals, including VEN. However, natural organic matter, surfactants, or co-contaminants in real wastewater can reduce its adsorption capacity through competitive interactions.^56–58^

#### 3.2.2 Biochar

Produced *via* pyrolysis of organic biomass, biochar is a sustainable alternative to AC and has attracted interest for its tunable surface characteristics. Surface activation using acids, alkalis, or oxidants can significantly enhance its capacity for VEN adsorption through improved surface area, pore volume, and the introduction of oxygen-containing functional groups.^48,59,60^

#### 3.2.3 Carbon-based nanomaterials

Carbon nanotubes (CNTs) and graphene oxide (GO) offer exceptional textural and surface chemical properties. Their π-conjugated frameworks facilitate π–π electron donor–acceptor interactions with aromatic rings in VEN, while surface functional groups enable hydrogen bonding. Functionalizing these nanomaterials can further enhance their specificity and loading capacity for VEN.^61–63^

#### 3.2.4 Metal–organic frameworks (MOFs)

MOFs are crystalline porous structures of metal ions coordinated to organic ligands, offering highly tunable porosity and surface chemistry. Their large surface area, tailored pore sizes, and potential for specific host–guest interactions render them attractive candidates for VEN removal. Nevertheless, their long-term stability in aqueous environments and sensitivity to pH fluctuations remain practical concerns.^64–66^

#### 3.2.5 Natural clays and zeolites

These aluminosilicate materials possess high cation-exchange capacities, structural microporosity, and modifiable surface properties. Modification techniques, such as surfactant intercalation or acid activation, have enhanced the organophilicity of VEN and improved its adsorption performance for VEN and other pharmaceuticals.^67–70^

#### 3.2.6 Chitosan-based composites

Have emerged as promising biosorbents for the removal of antidepressants from aqueous media due to their biocompatibility, biodegradability, and high affinity for polar pharmaceuticals. Chitosan, a natural polysaccharide derived from chitin, contains abundant –NH_2_ and –OH functional groups that facilitate electrostatic attraction, hydrogen bonding, and π–π interactions with antidepressant molecules such as venlafaxine and fluoxetine. When modified with materials such as graphene oxide, magnetite, or metal oxides, the surface area, porosity, and adsorption capacity of chitosan are significantly enhanced. For instance, magnetic chitosan–Fe_3_O_4_ composites enable efficient recovery after adsorption while maintaining high removal efficiency under neutral pH conditions. These interactions result in rapid adsorption kinetics and high removal efficiencies, as summarized in Table 2, where various chitosan-based biosorbents exhibit adsorption capacities ranging from 45 to 120 mg g^−1^ depending on surface modification and solution chemistry. Such performance highlights chitosan composites as sustainable, low-cost, and regenerable materials for the removal of pharmaceutical contaminants.^71–73^

**Table 2:** Summary of adsorbents used to remove venlafaxine (VEN) from aqueous solutions with key operational parameters

Adsorbent	Maximum adsorption capacity (mg g^−1^)	Optimal conditions	References
Activated carbon	35	pH 7, 25 °C	58
Biochar	2.5	pH 7.1, 24 °C	59
Functionalized CNTs	100	pH 7, 25 °C	74
Graphene oxide	19.72	pH 7, 25 °C	63
MOFs	420	pH 7, 25 °C	75
Modified zeolites	180	pH 7, 25 °C	76
Natural clays	15.3	pH 6.8, 25 °C	77
Chitosan-based composites	47	pH 6–7, 25 °C	78

**Fig. 3:**
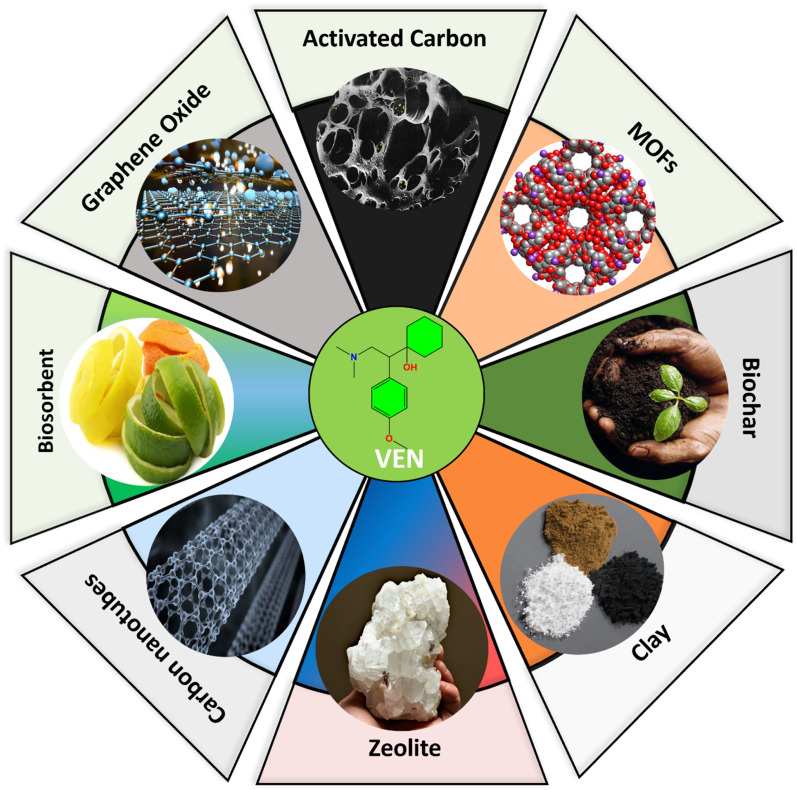
Interaction pathways and adsorption mechanisms of venlafaxine (ven) on different classes of adsorbents.

Numerous adsorbents have been investigated for the removal of venlafaxine (VEN) from aqueous media over the past decade. These materials range from conventional activated carbon and biochar to more advanced nanomaterials, including carbon nanotubes (CNTs), graphene oxide (GO), and metal–organic frameworks (MOFs). As shown in Table 2 and Fig. 3, these adsorbents rely on mechanisms such as π–π stacking, hydrogen bonding, electrostatic interactions, and pore filling. Notably, MOFs and functionalized CNTs exhibit the highest adsorption capacities, making them promising candidates for large-scale water treatment. The literature highlights that adsorbent performance is influenced by material surface area, pore structure, surface functionalization, and solution chemistry (*e.g.*, pH, ionic strength).^32,33,79–82^ A summary of selected adsorbents, their operational conditions, and VEN removal capacities is presented in Table 2. In conclusion, the choice of adsorbent for VEN removal depends on various factors, including surface functionality, porosity, environmental stability, regeneration potential, and economic feasibility. Table 2 and Fig. 3 illustrate a comparative schematic overview of the interaction mechanisms between VEN and various adsorbents.

#### 3.2.7 Covalent organic frameworks (COFs) as advanced Adsorbents for venlafaxine removal

Covalent Organic Frameworks (COFs) are an emerging class of crystalline, porous polymers constructed through strong covalent bonds between light elements such as C, H, N, O, and B. Their highly ordered structure, large surface area (often exceeding 1000 m^2^ g^−1^), and tunable pore architecture make them ideal candidates for the adsorption of complex organic contaminants, including antidepressants like venlafaxine (VEN). Unlike traditional adsorbents such as activated carbon or biochar, COFs offer pre-designable functionalities and uniform nanopores, allowing selective interaction with pharmaceuticals based on π–π stacking, hydrogen bonding, and electrostatic interactions.^83–86^

Although no direct studies yet report COF-based adsorption specifically for VEN, their proven performance in removing structurally similar pharmaceuticals such as diclofenac, carbamazepine, and fluoxetine strongly supports their applicability. The potential adsorption mechanism of VEN on COFs can be attributed to the synergistic effects of π–π electron donor–acceptor interactions between the aromatic rings of VEN and the COF framework, along with hydrogen bonding between amine groups of VEN and oxygen/nitrogen-containing sites on COFs. These interactions facilitate rapid mass transfer and high adsorption capacity, even under varying pH and ionic strength conditions.^85,87^

Future research should focus on tailoring COF structures with specific functional motifs or metal doping to improve affinity toward VEN and enhance regeneration performance. Overall, COFs represent a next-generation class of adsorbents with superior selectivity, chemical stability, and design flexibility for the removal of venlafaxine and related pharmaceutical pollutants.

### 3.3 Factors affecting adsorption

Various materials, including naturally occurring, synthetic, and hybrid adsorbents, have been investigated for VEN under different operating conditions, such as pH, adsorbent dose, and contact time. However, efforts are required to systematically analyze the wealth of studies to estimate operating conditions to achieve efficient VEN.

pH is one of the most critical process variables that can directly affect the uptake of La by adsorbents, as it influences the extent of VEN ionization and the surface characteristics of the adsorbent. VEN's ionization state and the adsorbent's surface charge are pH-dependent, influencing electrostatic interactions.^41,88–90^

#### 3.3.1 Temperature

The solution's temperature mainly affects the enlargement of adsorbents, the mobility of VEN ions, and the solid/liquid interface. Elevated temperatures can enhance adsorption capacity by increasing molecular mobility and pore diffusion rates. Thermodynamic parameters, along with temperature, were used to determine the nature of the adsorption process, including whether it is exothermic or endothermic, spontaneity, randomness, and whether the temperature is favorable for the process.^91,92^ The important thermodynamic parameters are Δ*G*°, Δ*H*°, and Δ*S*°, representing the change in Gibbs free energy, enthalpy, and entropy. The negative values of DG^0^ indicate that the adsorption process is spontaneous. Similarly, the positive values of Δ*H*^0^ suggest that the process is endothermic. Also, the magnitude of Δ*H*^0^ seems to be related to the type of sorption, VEN, physisorption (Δ*H*^0^ <50 kJ mol^−1^), and chemisorption (Δ*H*^0^ > 50 kJ mol^−1^). Moreover, the positive values of Δ*S*^0^ can be explained as an increase in entropy due to the exchange of metal ions by more mobile ions during the adsorption process.^90,93^

#### 3.3.2 Initial concentration

Higher initial concentrations of VEN can lead to increased adsorption until the adsorbent's saturation point is reached.^94,95^

#### 3.3.3 Contact time

Contact time significantly affects the adsorption process. It can also influence the economic efficiency of the process and the adsorption kinetics. Therefore, contact time is another performance-governing factor in the adsorption process. Adequate contact time ensures equilibrium, maximizing adsorption.^96–98^

#### 3.3.4 Presence of competing substances

Natural organic matter and other pharmaceuticals in water can compete for adsorption sites, potentially reducing the removal efficiency of VEN.

#### 3.3.5 Adsorbent dose

In general, the extent of adsorption of a solute increase with the increase in the concentration of an adsorbent because the increase in adsorbent concentration translates into increased active exchangeable adsorption sites. However, the fan adsorbent's overall solute adsorption per unit weight can decrease following the increase in adsorbent concentration due to interference caused by the interaction of active adsorbent sites.^99^

## 4 Advanced oxidation processes for antidepressant degradation

Advanced oxidation processes (AOPs) are a set of chemical treatment procedures designed to remove organic (and sometimes inorganic) materials in water and wastewater through oxidation reactions with hydroxyl radicals (˙OH) or sulfate radicals (SO_4_˙^−^). These radicals are highly reactive and non-selective, enabling the degradation of various pollutants, including pharmaceuticals like venlafaxine (VEN).

### 4.1 Conventional *vs.* advanced oxidation methods

The widespread occurrence of pharmaceutical residues such as venlafaxine (VEN) in aquatic ecosystems has emerged as a serious environmental concern, primarily due to their ecotoxicological risks and inherent resistance to natural attenuation processes.^22,100^ Conventional wastewater treatment technologies such as biological treatment, coagulation, flocculation, and sedimentation are generally ineffective in eliminating trace-level organic micropollutants, particularly those with high chemical stability and low biodegradability.^27,101^ For instance, VEN removal efficiencies in conventional activated sludge systems are frequently reported to be below 30%, highlighting the inadequacy of traditional methods in addressing such persistent contaminants.^102^

In response to these limitations, advanced degradation techniques, especially advanced oxidation processes (AOPs), have garnered significant attention due to their ability to achieve near-complete mineralization of recalcitrant pharmaceuticals. AOPs function by generating highly reactive species, most notably hydroxyl (˙OH) and sulfate (SO_4_˙^−^) radicals, that non-selectively oxidize a broad spectrum of organic pollutants into benign end products such as carbon dioxide and water.^103,104^ Various AOPs, including Fenton and Fenton-like reactions, photocatalysis, ozonation, and persulfate-based oxidation, have been successfully employed to target emerging contaminants such as VEN.^105–107^

Among these, persulfate-based AOPs, whether thermally activated or catalyzed by transition metal oxides, offer distinct advantages, including strong oxidative potential, operational versatility, and stability across diverse environmental conditions.^108^ These characteristics render them practical alternatives to classical Fenton systems, which are often constrained by narrow pH requirements and substantial sludge production.^109–111^ Moreover, incorporating heterogeneous catalysts, including metal oxide nanocomposites and graphene-derived frameworks, has substantially improved the reusability, selectivity, and overall degradation efficiency of AOPs for VEN and other pharmaceuticals. Table 3 illustrates Comparative Analysis of Conventional and Advanced Treatment Methods for Venlafaxine (VEN) Removal from Aqueous Media with Optimal Operating Conditions and Efficiencies.

**Table 3:** Comparative analysis of conventional and advanced treatment methods for venlafaxine (VEN) removal from aqueous media with optimal operating conditions and efficiencies

Method	Type	Conditions (pH, time, dose, *etc.*)	VEN removal efficiency	References
Activated sludge	Conventional	pH 7.0, HRT 8 h, no specific VEN dose; natural microbial degradation	∼20–30%	112
Coagulation–flocculation	Conventional	Alum dose 50 mg L^−1^, pH 6.5, settling time 30 min, VEN 1 mg L^−1^	<25%	27
Activated carbon adsorption	Conventional	pH 6.0, 60 min, 0.5 g L^−1^ adsorbent, VEN 10 mg L^−1^	∼70%	113
UV photolysis	Advanced	UV-C lamp (254 nm), pH 6.5, 30–60 min, VEN 10–20 mg L^−1^	60–75%	114
UV/H_2_O_2_ AOP	Advanced	pH 5.5, 0.2 mM H_2_O_2_, 30 min, VEN 10 mg L^−1^	>90%	115
Fenton process	Advanced	Fe^2+^ 0.1 mM, H_2_O_2_ 1 mM, pH 3.0, 60 min, VEN 5 mg L^−1^	∼92%	27
Persulfate/Fe_3_O_4_ catalyst	Advanced	pH 4.0, 1 mM persulfate, 0.2 g L^−1^ Fe_3_O_4_ catalyst, 45 min, VEN 10 mg L^−1^	∼95%	116
ZnO/UV photocatalysis	Advanced	pH 7.0, 0.5 g L^−1^ ZnO, UV exposure 60 min, VEN 20 mg L^−1^	∼96%	117

In conclusion, although conventional treatment methods remain integral for removing bulk organic matter and nutrients, the integration of AOPs into modern, hybrid treatment schemes is increasingly regarded as essential for the sustainable and efficient elimination of pharmaceutically active compounds, such as venlafaxine, from wastewater streams.

### 4.2 Types of AOP techniques for venlafaxine (VEN) removal

Advanced oxidation processes (AOPs) are versatile and highly effective techniques for degrading persistent pharmaceutical pollutants, such as venlafaxine (VEN). Due to its resistance to conventional treatment methods, VEN has been the subject of various AOP-based investigations, employing diverse oxidants and catalytic systems.

#### 4.2.1 Photocatalysis

The photocatalytic degradation of VEN utilizes semiconductors activated by light irradiation to generate reactive oxygen species (ROS), primarily hydroxyl radicals (˙OH). The most studied materials are titanium dioxide (TiO_2_) and graphitic carbon nitride (g-C_3_N_4_). Moreover, MoS_2_/g-C_3_N_4_ composites demonstrated 97.7% VEN degradation under simulated solar light, exhibiting pseudo-first-order kinetics and the formation of transformation products (TPs), indicating successful photodegradation and detoxification.^118^

#### 4.2.2 Photo electrocatalysis

Combining photocatalysis with electrochemical oxidation further enhances efficiency. Kallyni *et al.* reported the rapid degradation of VEN under real wastewater conditions, achieving 100% removal efficiency at UV/Ti/TiO_2_-ZIF-67, where the removal efficiency was influenced by catalyst loading, pH, and initial concentration.^65^ The improved performance was attributed to the formation of a *Z*-scheme heterojunction, which facilitated enhanced charge separation and reactive radical generation.

#### 4.2.3 Photo-assisted Fenton-like processes

The photo-assisted Fenton-like process represents an advanced hybrid oxidation strategy that integrates heterogeneous Fenton catalysis with UV or visible light irradiation to enhance the generation of reactive oxygen species (ROS), including hydroxyl radicals (˙OH) and sulfate radicals (SO_4_˙^−^). In conventional Fenton-like systems, the efficiency is often limited by the slow regeneration of Fe^2+^ from Fe^3+^ and by the narrow pH range (typically 2–4) required for optimal radical formation. The photo-assisted approach overcomes these limitations by photochemically reducing Fe^3+^ to Fe^2+^, thereby sustaining radical production even under near-neutral conditions.

Recent studies have demonstrated that visible-light-assisted Fenton-like systems effectively degrade persistent pharmaceuticals, including venlafaxine (VEN), through synergistic photocatalytic and redox mechanisms. For instance, Fe_3_O_4_@TiO_2_, CuFe_2_O_4_/graphene oxide, and CoFe_2_O_4_/BiOBr nanocomposites have shown remarkable performance due to enhanced electron transfer and minimized catalyst leaching.

The introduction of light irradiation significantly accelerates the redox cycling of Fe ions, promotes charge separation, and facilitates the degradation of aromatic intermediates of VEN into low-molecular-weight carboxylic acids and CO_2_. Furthermore, visible-light-driven sulfate radical systems (*e.g.*, Fe_3_O_4_@C activating peroxy mono sulfate, PMS) have shown enhanced VEN degradation efficiency with pseudo-first-order kinetics and mineralization degrees exceeding 90% within 60 minutes.^86,115,119,120^

Overall, the integration of photochemical and Fenton-like pathways offers superior oxidation potential, lower chemical consumption, and enhanced reusability of catalysts—making the photo-assisted Fenton-like process a promising route for the sustainable degradation of venlafaxine and other antidepressants in wastewater.

#### 4.2.4 Fenton and persulfate-based oxidation

Fenton-based oxidation uses Fe^2+^ and H_2_O_2_ to generate hydroxyl radicals through redox cycling, while persulfate activation produces sulfate radicals (SO_4_˙^−^), a more selective and longer-lived oxidant. A cysteine-assisted Fe^2+^/persulfate system enabled complete VEN degradation within 5 minutes under mild conditions, thanks to the accelerated Fe^3+^/Fe^2+^ cycle facilitated by cysteine, which sustained high ROS production across a broad pH range.^121–123^

#### 4.2.5 Direct photolysis and UV/H_2_O_2_

VEN is also susceptible to degradation under direct UV irradiation, with hydrogen peroxide addition significantly enhancing the process *via* ˙OH production. However, radical scavengers like tert-butanol markedly reduce degradation efficiency, reaffirming the dominant role of hydroxyl radicals.^111,115,124^

### 4.3 Reaction mechanisms and intermediates

VEN degradation through AOPs involves hydroxyl radical (˙OH) and sulfate radical (SO_4_˙^−^) pathways, with reaction mechanisms tailored to the oxidant type and the catalytic system employed.

#### 4.3.1 Hydroxyl radical (˙OH)-Driven mechanisms

Hydroxyl radicals are highly reactive, non-selective oxidants (*E*^0^ = +1.8 to +2.7 VNHE) that attack VEN through: radical addition to aromatic rings, hydrogen abstraction from aliphatic or phenolic groups, and electron transfer from nitrogen-containing moieties.

In photocatalysis, semiconductor excitation under light generates electron–hole pairs. The photogenerated holes (h^+^) oxidize surface-adsorbed water or hydroxide to ˙OH radicals:

1 h^+^ + H_2_O → ˙OH + H^+^

2 h^+^ + OH^−^ → ˙OH

In electrochemical AOPs, hydroxyl radicals form on the anode surface:

3 H_2_O → ˙OH + H^+^ + e^−^

In classical Fenton systems:

4 Fe(ii) + H_2_O_2_ → Fe(iii) + ˙OH + OH^−^

5 Fe(iii) + H_2_O_2_ → Fe(ii) + HO_2_˙ + H^+^

Ozone-based hybrid systems further produce ˙OH *via* chain reactions:

6 O_3_ + OH^−^ → HO_4_^−^

7 HO_4_^−^ → HO_2_˙ + O_2_˙^−^

8 O_2_˙^−^ + O_3_ → O_2_ + O_3_˙^−^

9 O_3_˙^−^ + O_2_ + O˙

10 O˙ + H_2_O → ˙OH + OH^−^

The ˙OH radicals initiate oxidative cleavage of the cyclohexanol moiety, demethylation of the dimethyl amino group, and subsequent ring opening, leading to intermediates such as hydro quinones and carboxylic acids. Final mineralization yields CO_2_, H_2_O, and inorganic ions.

#### 4.3.2 Sulfate radical (SO_4_˙^−^) driven mechanisms

Sulfate radicals (*E*^0^ = +2.6 to + 3.1 VNHE) are generated from peroxydisulfate (PDS) or peroxy mono sulfate (PMS) activation *via* heat, UV, transition metals, or alkaline conditions:

11 HSO_5_ → SO_4_^−^˙ + OH˙

12 S_2_O_8_^−2^ → 2 SO^−2^_4_˙

13 M^*n*+^ + HSO_5_^−^ → M^(*n*+1)+^ + SO_4_^−^˙ + OH^−^

14 M^*n*+^ + S_2_O_8_^−2^ → SO_4_^−^˙ + M^(*n*+1)+^ + SO_4_^−2^

SO_4_˙^−^ radicals selectively oxidize functional groups through: hydrogen abstraction from aliphatic side chains, Nucleophilic attack on nitrogen atoms, and π-system electrophilic attack on aromatic rings.

In VEN degradation, these radicals effectively demethylate the dimethyl amino group, oxidize the aromatic ether linkage, and facilitate ring fragmentation. Intermediates such as *N*-desmethyl-VEN, catechol derivatives, and short-chain dicarboxylic acids are typically observed before complete mineralization.

**Fig. 4:**
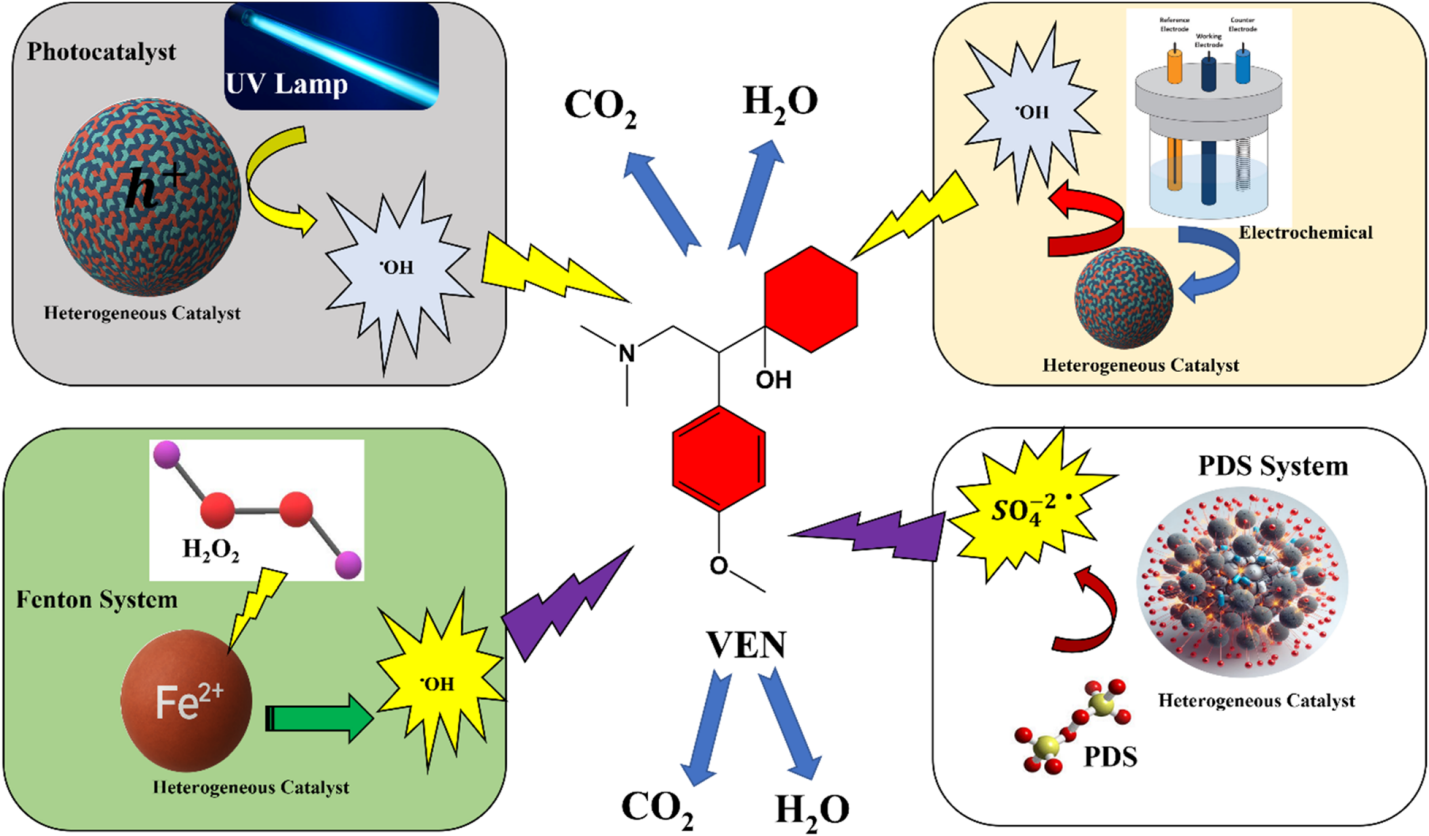
Reaction mechanisms and intermediates in advanced oxidation processes (AOPs) for venlafaxine degradation *via* hydroxyl and sulfate radical formation.

Moreover, the longer half-life and selective reactivity of SO_4_˙^−^ make them suitable for complex wastewater matrices, where competitive scavenging can hinder ˙OH-based systems. The reaction mechanism and intermediate are shown in Fig. 4.

The relationship between material structure and venlafaxine removal efficiency has become a focal point of recent studies. For adsorption-based systems, the surface oxygen functionalities, pore size distribution, and π-conjugated carbon structures directly influence π–π stacking, electrostatic, and hydrogen bonding interactions with VEN molecules.^48,125,126^ For instance, graphene oxide and functionalized CNTs display enhanced sorption capacity due to abundant carboxyl, hydroxyl, and epoxy groups that promote electron donor–acceptor interactions with the aromatic amine sites of VEN. Similarly, in advanced oxidation processes (AOPs), catalytic activity is largely governed by the redox potential and electron-transfer capability of metal centers or dopants. Transition metal oxides such as Fe_2_O_3_, MnO_2_, and Co_3_O_4_, when integrated into graphene frameworks or MOFs, enhance reactive oxygen species (ROS) generation through interfacial charge transfer, thereby accelerating hydroxyl (˙OH) and sulfate (SO_4_˙^−^) radical formation.^64,66,127^ These structure activity correlations reveal that the interplay between surface chemistry, electronic conductivity, and morphological stability is key to achieving superior degradation efficiency and reusability. The efficiency of AOP-based degradation and adsorption strongly depends on the structural and electronic configuration of the catalytic materials. Recent investigations revealed that bimetallic oxides and heterostructured composites exhibit superior catalytic performance due to enhanced charge separation, oxygen vacancy formation, and synergistic redox cycling between metal centers. For instance, coupling Fe–Cu or Mn–Co oxides on graphene or biochar frameworks not only accelerates radical generation (˙OH, SO_4_˙^−^) but also stabilizes the catalyst surface during repeated use.^128^ The activity of these materials can be correlated with their band gap energy and surface electron density, which govern both adsorption affinity and oxidative degradation kinetics. Such structure–activity correlations are crucial for designing next-generation heterogeneous catalysts with tunable selectivity and reusability.^66,129,130^

## 5 Comparative analysis: adsorption *vs.* advanced oxidation processes

The removal of emerging pharmaceutical pollutants such as venlafaxine (VEN) from aquatic environments has prompted the development of both adsorptive and oxidative treatment strategies. This section presents a comparative analysis of these techniques, evaluating their removal efficiency, mechanisms, operational costs, reusability, and environmental impact, and highlighting their advantages and limitations as illustrated in Table 4.

**Table 4:** Comparative analysis of these techniques, evaluating their removal efficiency, mechanisms, operational costs, reusability, and environmental impact, and highlighting their advantages and limitations

Parameter	Adsorption	Degradation (AOPs)	References
Removal efficiency	Moderate to high (60–90%)	High (>90)	131–134
Mechanism	Physical/chemical binding	Oxidation by radical	135 and 136
By product risk	None	High (TPs may be toxic)	135 and 136
Cost and setup	Low-cost, simple equipment	Higher cost, complex setup	137 and 20
Reusability	High with regenerable adsorbents	Moderate (catalyst fouling possible)	138–140
Environmental impact	Waste phase transfer	Risk of incomplete mineralization	141–143

### 5.1 Removal efficiency

Advanced degradation technologies, particularly Advanced Oxidation Processes (AOPs), have demonstrated superior removal efficiencies, often exceeding 90%, by achieving near-complete mineralization of VEN into benign end-products, such as CO_2_ and H_2_O.^131,132^ In contrast, adsorption processes rely on physical or chemical interactions, and while effective, their efficiencies are contingent on the adsorbent's surface area, porosity, and functional groups.^133,134^

### 5.2. Mechanism and byproducts

Adsorption operates primarily through surface interactions and does not chemically alter the target pollutant, thereby minimizing the risk of secondary pollution.^135^ Conversely, degradation processes such as photocatalysis, Fenton, or persulfate-based oxidation involve radical-driven chemical transformation of VEN. Although effective, they may yield transformation products (TPs) like hydroxylated or demethylated derivatives, some of which exhibit residual ecotoxicity.^136^

### 5.3 Operational cost and complexity

Adsorption is favored for its cost-effectiveness and operational simplicity, with minimal energy requirements and straightforward implementation.^137^ However, the regeneration or disposal of saturated adsorbents introduces an additional operational burden. On the other hand, degradation processes, especially UV-activated AOPs or electrochemical oxidation, require higher energy input, specialized reactors, and catalyst dosing, elevating both capital and operational costs.^20^

### 5.4 Reusability and sustainability

Many adsorbents, such as biochar, activated carbon, and magnetic nanocomposites, can be regenerated and reused multiple times, offering a sustainable route for VEN removal.^138,139^ Degradation catalysts, including TiO_2_ or metal-doped oxides, may undergo deactivation due to surface fouling or photo-corrosion, which limits their long-term efficiency and requires frequent replacement or surface modification.^140^

### 5.5 Environmental impact

While adsorption involves phase transfer of VEN, necessitating safe disposal of loaded adsorbents, it avoids the formation of unknown byproducts.^141,142^ In contrast, degradation strategies aim to eliminate VEN; however, if incomplete, they may release toxic intermediates into the environment, necessitating thorough toxicity assessments before large-scale application.^143^

## 6 Current challenges and research gaps

Despite notable advances in removing venlafaxine (VEN) from aqueous systems through adsorption and advanced oxidation processes (AOPs), several critical challenges and research gaps remain that limit the full-scale implementation and sustainability of these approaches.

### 6.1 Incomplete mineralization and toxic transformation products (TPs)

While AOPs effectively degrade VEN, forming intermediate transformation products, some of which exhibit higher toxicity than the parent compound, raising environmental and regulatory concerns. The lack of comprehensive identification and ecotoxicological evaluation of these TPs remains a significant knowledge gap that must be addressed through advanced analytical techniques such as LC-MS/MS and high-resolution mass spectrometry.

### 6.2 Limited selectivity and interference by Co-contaminants

In real wastewater matrices, competing organic and inorganic substances can hinder the selectivity and efficiency of both adsorptive and oxidative processes. Research on the synergistic or antagonistic effects of co-contaminants on VEN removal is currently limited and requires systematic exploration under environmentally relevant conditions.

### 6.3 Catalyst and adsorbent stability

Catalyst deactivation (*e.g.*, photo-corrosion in photocatalysis or iron sludge accumulation in Fenton-like processes) and adsorbent saturation pose operational bottlenecks. Enhancing the structural and chemical stability of nanocomposites and optimizing regeneration protocols without compromising performance are ongoing challenges.

### 6.4 High energy and resource demand

Several AOPs, particularly those involving UV or electrochemical activation, suffer from high energy consumption, limiting economic viability. Integrating renewable energy sources or developing visible-light-active catalysts offers a promising, yet underutilized, pathway toward sustainability.

### 6.5 Lack of standardization and real-world validation

Most studies use synthetic water matrices under idealized lab-scale conditions. Standardized protocols and long-term pilot studies using real wastewater are needed to evaluate performance consistency, scalability, and environmental safety.

### 6.6 Life cycle and techno-economic assessments

Few studies have conducted comprehensive life cycle assessments (LCA) or techno-economic analyses (TEA) of VEN removal technologies. These are crucial for evaluating the environmental footprint and economic feasibility of integrating these methods into existing wastewater treatment frameworks.

## 7 Future perspectives

Future research on venlafaxine (VEN) removal should focus on the development of integrated, selective, and sustainable treatment systems that address operational efficiency and environmental safety. Hybrid technologies that combine adsorption with AOPs, such as adsorption photocatalysis or magnetic biosorbent Fenton systems, represent a promising avenue, offering synergistic benefits in removal efficiency and catalyst reusability.

The design of novel materials, including multifunctional nanocomposites and bio-based hybrid frameworks, should prioritize enhanced selectivity toward pharmaceuticals, stability under variable wastewater conditions, and facile regeneration. Special attention should be given to materials that operate efficiently under visible light and at neutral pH, thereby reducing energy input and chemical consumption.

Furthermore, a paradigm shift toward real wastewater testing is necessary to validate lab-scale findings and ensure scalability. This includes conducting long-term pilot studies, evaluating performance in the presence of complex pollutant mixtures, and performing comprehensive toxicity assessments of transformation products (TPs) to ensure that the degradation pathway leads to environmentally benign outcomes.

Despite the substantial progress in developing bimetallic and heterostructured metal oxide catalysts, several challenges remain unresolved. Catalyst deactivation due to surface fouling, metal leaching, and reduced redox efficiency during cyclic operation continues to limit their practical deployment. Additionally, many reported studies rely on simulated laboratory conditions that may not fully represent the complexity of industrial wastewater systems. Future research should focus on designing catalysts with enhanced electron transport efficiency, sustainable synthesis methods, and high tolerance to real matrix interference. Integrating adsorption, photocatalysis, and Fenton-like oxidation within hybrid systems could provide new routes for simultaneous pollutant degradation and catalyst regeneration. Furthermore, coupling density functional theory (DFT) and machine learning could accelerate the prediction of active sites and reaction pathways, thus bridging the gap between mechanistic understanding and large-scale application.

## 8 Conclusion

The effective removal of venlafaxine (VEN) from contaminated water bodies demands innovative approaches beyond traditional treatment systems. This review highlights that adsorption remains a reliable, low-cost, and environmentally sustainable method, especially when leveraging bio-based adsorbents and industrial wastes within a circular economy model. However, adsorption alone is insufficient for complete degradation, often limited to surface–level interaction without mineralization. In contrast, advanced oxidation processes (AOPs) such as persulfate and photocatalysis exhibit high efficacy in degrading recalcitrant pharmaceutical residues, producing fewer toxic byproducts and achieving complete mineralization. Combining tailored nano catalysts and optimized operational parameters, pH, catalyst dosage, and oxidant loading have proven critical in maximizing efficiency. For real-world applications, hybrid systems integrating adsorption as a pre-treatment followed by AOP based mineralization emerge as a promising, scalable solution. Future research should prioritize continuous-flow studies, real wastewater matrices, and cost–benefit analysis to transition from laboratory-scale feasibility to full-scale implementation.

## Conflicts of interest

There are no conflicts to declare.

## Data Availability

This work does not include any available data as it is a review article.
